# The determinants of survival among adults with cystic fibrosis—a cohort study

**DOI:** 10.1186/s40101-021-00269-7

**Published:** 2021-11-08

**Authors:** Magdalena Durda-Masny, Joanna Goździk-Spychalska, Aleksandra John, Wojciech Czaiński, Weronika Stróżewska, Natalia Pawłowska, Jolanta Wlizło, Halina Batura-Gabryel, Anita Szwed

**Affiliations:** 1 Institute of Human Biology and Evolution, Faculty of Biology, Adam Mickiewicz University, Uniwersytetu Poznanskiego 6 St, 61-614 Poznan, Poland; 2 Department of Pulmonology, Allergology and Respiratory Oncology, Poznan University of Medical Sciences, Szamarzewskiego 82/84, 60-569 Poznań, Poland

**Keywords:** Cystic fibrosis, Lung function, Life expectancy, BMI, *Pseudomonas aeruginosa*

## Abstract

**Background:**

Cystic fibrosis (CF) is one of the most common autosomal recessive diseases. Factors contributing to disease exacerbations and survival rate of CF patients are type of mutation in the *CFTR* gene, poor nutritional status, lung failure, and infection development by *Pseudomonas aeruginosa*. The study aimed to evaluate the relationship between the severity of mutation, nutritional status, lung function, and *Pseudomonas aeruginosa* prevalence and survival rate in adult patients with cystic fibrosis.

**Methods:**

A study of 124 (68 ♀ and 56 ♂) adults with CF aged 18–51 years were evaluated for (a) type of mutation in the *CFTR* gene, (b) nutritional status (BMI), (c) lung function (FEV_1_%), and (d) *Pseudomonas aeruginosa* prevalence. For statistical calculations, Kaplan-Meier analysis of survival, chi-squared test for multiple samples, and logistic regression were used.

**Results:**

The type of mutation (*χ*^2^ = 12.73, df = 3, *p* = 0.005), FEV_1_% (*χ*^2^ = 15.20, df = 2, *p* = 0.0005), *Pseudomonas aeruginosa* prevalence (*χ*^2^ = 11.48, df = 3, *p* = 0.009), and BMI (*χ*^2^ = 31.08, df = 4, *p* < 0.000) significantly differentiated the probability of survival of patients with CF. The shortest life expectancy was observed in patients with a severe type of mutation on both alleles, FEV_1_% < 40, subjects in whom *Pseudomonas* culture was extensively drug-resistant or pandrug-resistant, and patients whose BMI was lower than 18.5 kg/m^2^. The period from 30 to 40 years of age was the most critical in CF adults’ lifespan. The risk of adults with CF death doubled with *Pseudomonas aeruginosa* prevalence (OR = 2.06, 95% CI 1.29; 2.28) and eightfold when the bacteria acquired antibiotic resistance (OR = 8.11, 95% CI 1.67; 38.15).

**Conclusions:**

All factors included in the study were significantly related to the survival rate of patients with cystic fibrosis.

## Introduction

Cystic fibrosis (CF) is one of the most common autosomal recessive diseases caused by 2102 mutations of the *CFTR* gene (http://www.genet.sickkids.on.ca/StatisticsPage.html). The type of mutation is considered as one of the most important factors determining the survival rate. Severe mutations of at least one allele reduce the survival of patients with CF and are associated with lung function deterioration [1]. It is already known that the other factors contributing to disease exacerbations are lung failure and infection development [2]. One of the most important pathogens in adults with CF, causing chronic infection and evolve into antibiotic resistance, is *Pseudomonas aeruginosa*. Previous studies showed that *P. aeruginosa*, mainly multidrug resistance culture, is associated with morbidity and mortality in patients with CF [3]. The decline in lung function is also associated with poor nutritional status [4–8]. Lung function, nutritional status, and the presence of *P. aeruginosa* are interrelated, and the studies by John et al. [9] have shown that decreased lung function (FEV_1_% < 40), undernutrition (BMI < 18.5), and severe mutation type are associated with a higher probability of *P. aeruginosa* acquisition and with the higher level of its resistance to antibiotic treatment.

Until now, research on the factors affecting the survival rate of patients with CF has focused mainly on the pediatric population. Due to advances in medical care, the number of adults with CF is steadily growing [10]. A multicenter report on patients with CF at the age of 40 and above, conducted by Hodson et al. [11], showed that a significant number of patients reach the age of 40 or more. However, the answer to the question of why some CF patients live longer than others is still unclear, which, together with the increasing number of adult patients, generates the need for research on the factors affecting the survival rate in this group. Hence, the study aimed to evaluate the relationship between lung function, *P. aeruginosa* prevalence, nutritional status, and the severity of mutation type and survival rate in adult patients with CF.

**Table 1:** The number of patients in each age cohort at the beginning of the study and the number of patients who died during the course of the study

Patients’ age cohorts at the beginning of the study	Number	Percent	Number of patients who died during the study
18–23	60	48.39	8
24–29	35	28.23	8
30–35	22	17.74	3
36–41	7	5.64	2

## Methods

The study we conducted was a cohort study in its nature. The same group of patients recruited for the research in 2010 was subjected to annual examinations for the next 9 years. At the beginning of the study, the cohort consisted of 124 patients aged 18 to 41 years old, treated at the Department of Pulmonology, Allergology, and Respiratory Oncology, Poznan University of Medical Science, in Poland. The group consisted of 68 women and 56 men. For the period of 9 years, i.e., until 2019, the values of respiratory parameters, nutritional status, the presence of *P. aeruginosa*, and the degree of its resistance to antibiotics were analyzed in the studied group, at least annually for each patient. A total of 21 people died during the research, and it was the only reason why the size of the study group decreased over time (Table 1). The complete data was obtained from all other patients included in the study. Statistical analyses were performed on the last measurements obtained at the end of the study for living patients, or on the last measurements obtained before the death, for patients who died during the study.

Adults with CF who either underwent a lung transplant, were pregnant, smoked, used systemic glucocorticosteroids, or had pulmonary exacerbation during 4 weeks preceding the study were excluded.

Information about the type of mutation, nutritional status, lung function, and *P. aeruginosa* prevalence was collected. Data about the type of mutation of the *CFTR* gene were obtained from the archives of medical records of the Department of Pulmonology, Allergology, and Respiratory Oncology of the University of Medical Sciences in Poznan. To systematize the type of mutation present in each patient, all subjects were divided into four groups based on severity, in accordance with the widely accepted mutation classification in the *CFTR* gene [12, 13] (Table 2): (1) patients with severe types of mutation (I, II, III mutation classes) on both alleles (I–III/I–III), (2) heterozygous patients with a severe type of mutation on one allele and mild (I–III/IV–V) or unclassified mutation (other mutations, including those unknown) on another allele (I–III/u), (3) patients with mild types of mutation (IV and V mutation classes) on both alleles (IV–V/IV–V), and (4) unclassified mutations (u/u) (Table 3).

**Table 2:** Classification of the *CFTR* gene mutations (Lubamba et al. [12]; De Boeck et al. [13])

Class	Consequences	List of mutations attributed
I	CFTR is not synthesized because of stop codons or splicing defects.	G542X, W1282X, R553X, 3950delT
II	CFTR is synthesized but in an immature and is mostly degraded by the ubiquitin-proteasomal pathway.	F508del, N1303K
III	CFTR is synthesized and transported to the plasma membrane, but its activation and regulation by ATPor cAMP are disrupted.	G551D, G178R, S549N, S549R, G551S, G970R, G1244E, S1251N, S1255P, G1349D
IV	CFTR is synthesized and expressed at the plasma membrane, but chloride conductance is reduced.	R334W, G314E, R347P, D1152H
V	CFTR synthesis or processing is partly defective.	3849+ 10 kb C→T, 3272-26 A→G, 2789+5G→A
Unclassified		All other mutations, including those unknown

**Table 3:** Number and percentage of adult CF people in the category of *CFTR* mutation, nutritional status, lung function, and *Pseudomonas aeruginosa* infection

Variable	Number	*N* (%)
*Genotype*		
I–III/I–III	40	32.26
I–III/IV–V or I–III/u	31	25.00
IV–V/IV–V	24	19.35
u/u	29	23.39
*FEV*_*1*_*%*		
FEV_1_% > 70	31	25.00
FEV_1_% 70–40	47	37.90
FEV_1_% < 40	46	37.10
*Pseudomonas aeruginosa*		
*Pseudomonas* culture-negative	36	29.03
Non-MDR	37	29.84
MDR	24	19.36
XDR/PDR	27	21.77
*Nutritional status*		
BMI ≥ 25	5	4.03
BMI 18.5–24.9	74	59.68
BMI 17–18.49 (class II malnutrition)	24	19.35
BMI 16–16.99 (class I malnutrition)	9	7.26
BMI < 16 (emaciation)	12	9.68

*I–III/I–III* patients with severe types of mutation on both alleles, *I–III/IV–V or I–III/u* patients with a severe type of mutation on one allele and mild or unclassified mutation on another allele, *IV–V/IV–V* patients with mild types of mutation on both alleles, *u/u* patients with unclassified mutations on both alleles, *BMI* body mass index, *FEV*_*1*_*%* forced expiratory volume in one second, *Non-MDR* non-multidrug-resistant patients, *MDR* multidrug-resistant patients, *PDR+XDR* pandrug-resistant and extensively drug-resistant patients

Lung function was determined by a spirometry test using a diagnostic Jaeger MasterScreen system (Erich Jaeger GmbH; Würzburg, Germany). Data about predicted forced expiratory volume in 1 s (FEV_1_%) were collected from 124 patients. According to the Cystic Fibrosis Trust [14], all subjects were divided into three subgroups based on FEV_1_%: (1) patients within the norm (FEV_1_% > 70), (2) with moderate pulmonary impairment (FEV_1_% 70–40), and (3) severe pulmonary impairment (FEV_1_% < 40) (Table 3).

The microbiological examination carried out by a microbiological laboratory was performed in all patients. Microbiological data allowed us to classify patients into the following groups (Table 3): (1) *Pseudomonas* culture-negative and (2) *Pseudomonas* culture-positive. Drug susceptibility was measured using the Eucast v.6.0 method. *Pseudomonas* culture-positive patients were divided into the following: 2a, patients in whom all antibiotics used to treat infections caused by bacterial colonization were fully effective (non-multidrug resistant/ non-MDR); 2b, subjects in whom *Pseudomonas* culture was insensitive (resistant or moderately sensitive) to at least one antibiotic from at least three groups of antibacterial drugs (multidrug-resistant (MDR)); and 2c, patients in whom *Pseudomonas* culture was extensively drug-resistant (XDR) or pandrug-resistant (PDR). The above division was made by the definitions from the work of Magiorakos et al. [15].

Nutritional status was determined based on body mass index (BMI) that was calculated by dividing body weight by height squared (kg/m^2^). To obtain this data, anthropometric measurements were taken. The body height was measured without shoes and in underwear, with a GMP anthropometer, with a measurement accuracy of 1 mm. Body weight was measured using a medical scale with a measurement accuracy of 100 g. To exclude measurement errors, all measurements were performed by one experienced researcher. Based on the BMI, a group of adults with CF was divided into five (Table 3): emaciation (BMI < 16), class II undernutrition (BMI = 16–16.99), class I undernutrition (BMI = 17–18.49), within the norm (BMI = 18.5–24.9), and overweight (BMI ≥ 25).

The study was performed with the approval of the local research ethics committee (resolution No. 51/17). All participants had provided their written informed consent of participation in this study.

The effect of nutritional status, lung function, *P. aeruginosa* prevalence, and the severity of mutation type on survival was determined with the Kaplan-Meier method. Differences in the survival rate within the study groups were assessed with the chi-squared test for multiple samples. To determine the risk of death depending on the type of mutation, nutritional status, lung function, and the impact of *P. aeruginosa* on survival rate, logistic regression was used. *p*-values < 0.05 defined statistically significant differences. Statistical analysis was performed with the Statistica 12.0 data analysis software system (StatSoft Polska).

## Results

**Fig. 1:**
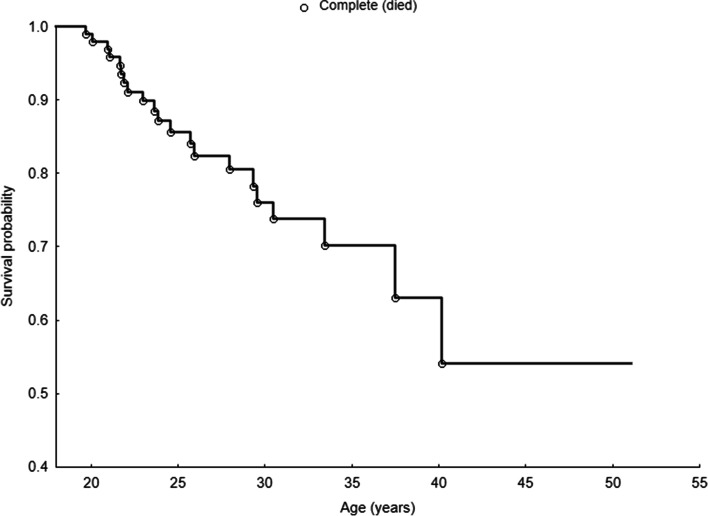
Kaplan-Meier plot for the age of patients with cystic fibrosis

Survival analysis using the Kaplan-Meier method has shown that the survival of patients with CF stood at 100% up to the age of 20; then, it started to decline gradually (Fig. 1). The decline in the probability curve showed that almost 27% of patients will not exceed the age of 30 years of life, and almost 48% of patients will not exceed the age of 40.

In our study group, all patients who lived to the age of 40 are still alive (*N* = 7). At the time of writing this article, three subjects were 41 years old, and four were 42, 43, 49, and 51 years old.

**Fig. 2:**
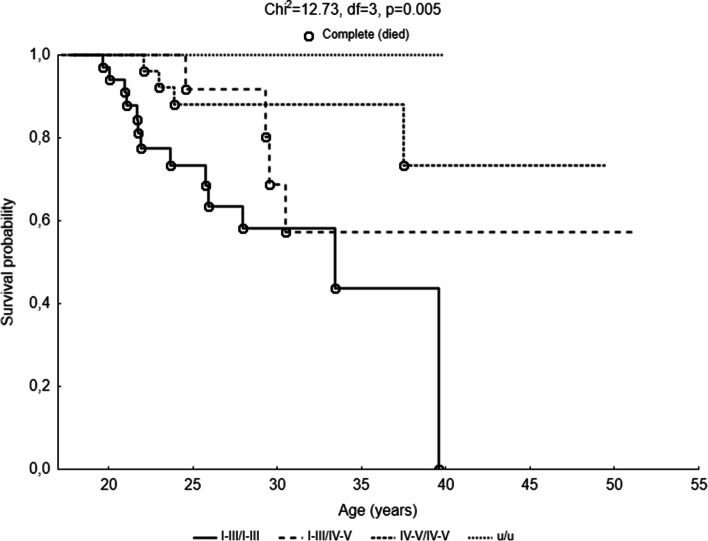
Probability functions depicting the age of patients with cystic fibrosis in categories of mutation

The probability of survival was different depending on the severity of the mutation type (*χ*^2^ = 12.73, df = 3, *p* = 0.005). The average life expectancy of patients with I–III/I–III mutations was the lowest. The survival curve (Fig. 2) in this group decreased after the age of 20, and the probability of death before the age of 35 was almost 60%. In this group, there were no people over 40 years of age. Subjects with I–III/IV–V or I–III/u mutations lived longer than in the I–III/I–III group. The survival curve in this group began to decline after the age of 23 years, and the probability of death before reaching the age of 32 was over 40%. It is seen that the curve stabilized after the age of 32, and it is worth mentioning that there was one person in this group who has reached the age of 49. Among patients with I–IV/I––IV mutations, the decline in the survival curve was small, and the curve stabilized after 25 years of age. However, it can be seen that the 40th year of life was critical for their survival, since only three patients managed to pass over this age. In the group with u/u mutations, there were no cases of death recorded, and the survival curve was stable. Two patients from this group were over the age of 40, and one was over the age of 50 (Fig. 2).

**Fig. 3:**
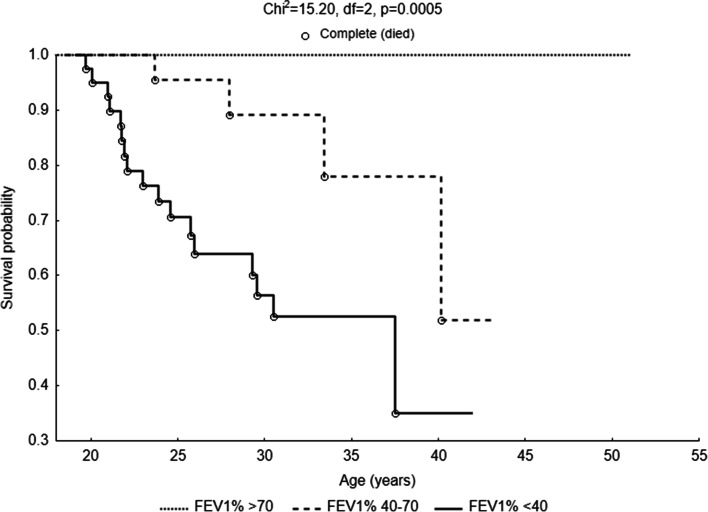
Probability functions depicting the age of patients with cystic fibrosis in categories of lung function (FEV_1_%)

The factor that significantly differentiated patients in terms of life expectancy was also lung function (*χ*^2^ = 15.20, df = 2, *p* = 0.0005). Those patients with CF whose FEV_1_% > 70% lived longest. The mortality rate was equal to 0, and additionally, there were people over 40 and 50 years old in this group. The survival curve of CF adults with FEV_1_% between 40 and 70% was steeper. The chance for those patients to reach the age of 40 was in the range of 50%. Approximately 20% will die before the age of 34 and another 28% of patients before reaching the age of 40. The lowest life expectancy applied to patients with FEV_1_% < 40. The probability of survival in this group began to decrease after reaching the age of 20. The survival curve was very steep up to the critical point of 26 years of age. In this group, the probability of death before the age of 30 was almost 50%, and only about 35% of patients have a chance to live to be 40 years old (Fig. 3).

**Fig. 4:**
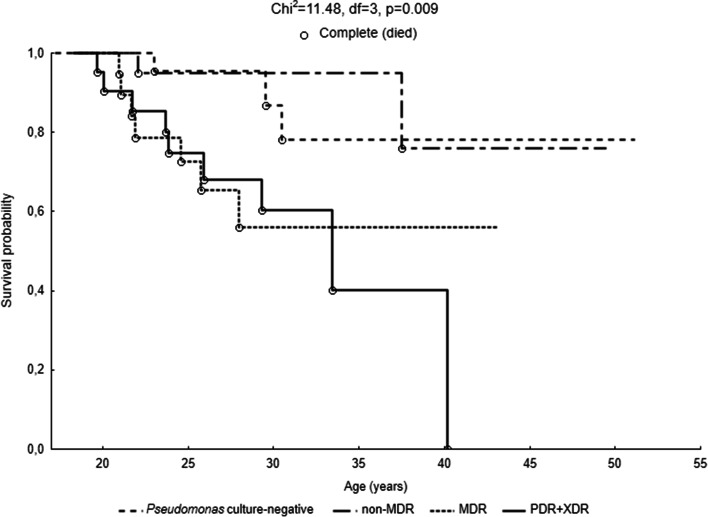
Probability functions depicting the age of patients in categories of *Pseudomonas aeruginosa* infection and antibiotic resistance (*P. aeruginosa* 4 categories)

**Fig. 5:**
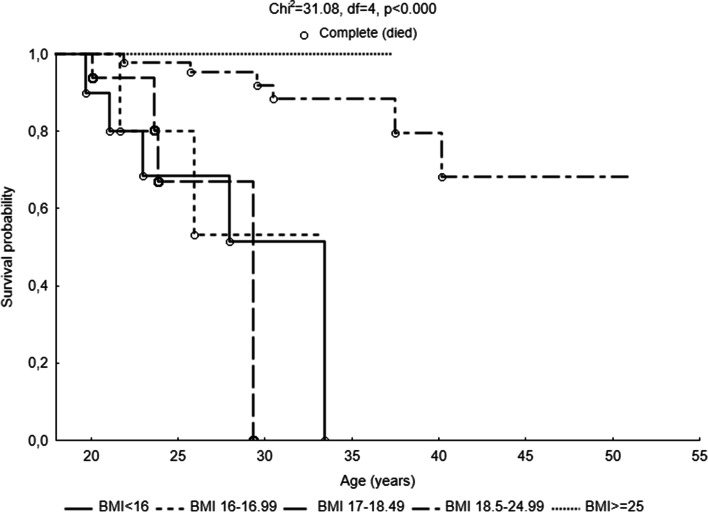
Probability functions depicting the age of patients with cystic fibrosis in categories of nutritional status (BMI)

Kaplan-Meier survival analysis showed also that *P. aeruginosa* significantly influenced the length of the patient’s life (*χ*^2^ = 11.48, df = 3, *p* = 0.009). The longest average life expectancy was indicated among *Pseudomonas* culture-negative and non-MDR patients (Fig. 4). The survival curve was comparatively stable in both groups. The probability of exceeding 40 years of age in these patients was approximately 80%. The average life expectancy was lower in MDR patients. The probability of survival was decreasing after 22 years of life, and the probability of exceeding the age of 30 was less than 60%. The survival of XDR and PDR patients was the most detrimental. The probability of survival was decreasing steadily from the age of 20 up to the age of 40. Furthermore, nutritional status assessed by BMI (Fig. 5) significantly influenced the length of patients’ life (*χ*^2^ = 31.08, df = 4, *p* < 0.000). There were no deceased among overweight patients, and their survival curve was stable. The survival curve of the properly nourished patients (BMI 18.5–24.99 kg/m^2^) was characterized by a slight decline of up to the age of 30. The highest decrease occurred between the age of 30 and 40. In a group of adults with CF within the norm, the life span exceeded 50 years. In turn, in all groups of undernourished subjects, the survival curve dropped significantly after reaching the age of 20. The life expectancy of the respondents in these groups did not exceed 34 years of age. The survival curves of all three subgroups were steep. All patients with BMI < 16.00 died before reaching the age of 30. The probability of survival among emaciated adults was significantly lower than among those from the I and II classes of undernutrition.

The results of logistic regression analysis showed that the risk of death increased almost sixfold when FEV_1_% fell below normal (OR = 5.83, 95% CI 2.51; 13.52, *p* < 0.001), more than four times when BMI fell below normal (OR = 4.17, 95% CI 1.65; 10.50, *p* = 0.002), and twice in the case of having a severe *CFTR* gene mutation (OR = 2.35, 95% CI 1.17; 4.71, *p* = 0.01). The risk of death for a patient with CF increased twice with *P. aeruginosa* prevalence (OR = 2.06, 95% CI 1.29; 2.28, *p* = 0.002) and eight times if the bacteria acquired antibiotic resistance (OR = 8.11, 95% CI 1.67; 38.15, *p* = 0.008).

## Discussion

The probability of survival among adults with CF is a result of both genetic and environmental factors contributing to the course of the disease. Our research is one of the first to show the impact of the factors such as the severity of mutation in the *CFTR* gene, nutritional status, lung function, and *P. aeruginosa* prevalence on survival rate in adult patients with cystic fibrosis. In our study, group survival curve started to decrease after 20 and became stable after 40 years of age. Most of the deaths took place in the period between the age of 30 and 40 years. The fundamental factor contributing to the average life expectancy was the type of mutation. Since the early nineties of the twentieth century, mutation causing CF has been categorized into five classes based on molecular mechanisms of CFTR chloride channel dysfunction [16], which corresponds to the phenotype of the disease. Our research indicated the difference in life expectancy between the groups of patients with CF distinguished according to the severity of the mutation. Patients with a mild mutation on one allele or both alleles lived longer than subjects from the group with severe types of mutation on both alleles. Similar results have been shown by McKone et al. [17], who found that the mortality rate was lower among patients with IV and V mutation classes compared to II class (F508del homozygotes). Currently, ongoing molecular studies concerning cell and gene therapy are a milestone in CF treatment, as the new strategies of therapy allow transferring the wild-type *CFTR* gene to the airway cells [18]. Apart from gene therapy, an important role is played by the early determination of the *CFTR* gene mutation type then followed by a prompt implementation of modern treatment methods. Neonatal screening allows for early diagnosis and therefore contributes to the better growth of patients and their better pulmonary outcomes [19]. Based on statistics, a patient with CF that gets a proper treatment and undergoes an adequate medical care that corresponds to the latest knowledge regarding the effects of a given mutation, at the early stage of disease, has a greater chance for a longer life compared to patients who lived in previous decades. Our study showed, however, that independent of other factors, a strong influence of the genetic factor on the rate of disease progression is still observed. The most critical life period in adults with CF characterized by a severe type of mutation falls within a period between the ages of 20 and 30 years. Similarly, for I–III/IV–V, patients’ crucial stage of the disease occurs between the ages of 25 to 31. It can be therefore summed up that the strongest impact of *CFTR* mutation in a CF patients’ life expectancy is being observed from the age of 20 up to about 31. This suggests that in adult CF patients, the type of mutation may still be the most important predictor for the prognosis of the disease. However, in our studies, some patients who shared the same type of mutation had a different disease severity. Taking this fact into account, the non-genetic factors that contribute to the survival rate of patients should not be underestimated [20, 21]. Our results indicate that the reduction of the value of each of the analyzed non-genetic variables below the specified threshold, i.e., FEV1% < 40%, BMI < 18.5, and XDR or PDR *Pseudomonas* prevalence, entails a number of negative consequences inevitably leading to the patient’s death. Therefore, when predicting CF patient’s survival, each of the variables analyzed in these studies should be taken into account.

The maintenance of proper lung function and prevention of undernutrition is considered as a priority in CF treatment. These factors provide the basis for keeping a patient in a state of stability. The results of this and previous studies [22] confirm the impact of lung function and nutritional status on the survival of adults with CF, showing that severe and moderate pulmonary impairment has a major impact on average life expectancy. Critical moments concern exclusively adults with FEV_1_% < 70%. Our results are consistent with the findings of other authors [23, 24]. The significance of lung function, considered as an important factor contributing to CF patients’ mortality, has been emphasized already by many researchers [25, 26]. Henry et al. [27] after performing stepwise regression showed that lung function described by FEV_1_% has the main effect on a patient’s survival. According to Belkin et al. [28], FEV_1_% < 30% increases the risk of death among both adult and pediatric patients with CF. Similarly, our analysis showed that FEV_1_% < 40% is critical for patients’ mortality, making it almost impossible for them to live into the age of 40. Furthermore, in a group of patients with severe pulmonary impairment, the majority of subjects die before the age of 30. Effective pulmonary disease therapy can, therefore, form the basis for preventing exacerbations and contribute to better survival of a patient with CF. Our probability of survival analysis highlights the problem of serious pulmonary exacerbations occurring between the age of 20 and 40. The survival curve for patients after the age of 40 was mostly stable. A study by Simmonds et al. [23] comparing adult patients with CF aged ≥ 40 with those who died before the age of 30 showed also that patients without any respiratory disease and with higher BMI were more likely to live into their 40s.

Another factor affecting the course of lung diseases are pathogen infections. Among adults with CF, *P. aeruginosa* is the most frequently occurring pathogen, affecting approximately 80% of the patients [29]. Our studies showed that the presence of *P. aeruginosa* decreases average life expectancy. Previous studies conducted among children confirm the adverse effect of *P. aeruginosa* on survival, lung function, and weight percentiles [30]. The consequences of *P. aeruginosa* presence in adults are not only similarly negative but also tend to exacerbate. Courtney et al. [26] showed that *P. aeruginosa* infection significantly affects CF adults’ mortality. Their studies revealed that the majority of patients who died (98%) had a chronic infection caused by this pathogen. We showed that the most critical point in survival of CF patient is when *P. aeruginosa* becomes fully or partly resistant to antibiotic treatment. Lechtzin et al. [29] obtained similar results, confirming that multiple antibiotic-resistant *P. aeruginosa* is associated with FEV_1_% reduction and pulmonary disease.

Moreover, exacerbations in lung function may severely affect the nutritional status of people with CF. Our study showed very explicit differences in the probability of survival with nutritional status. A significant number of patients in a group with a BMI < 18.5 died before even turning the age of 30. Undernourished adults with CF lived no longer than 34 years old. Undernutrition is to a large degree a consequence of pancreas insufficiency, fat malabsorption, micronutrient deficiencies, and increased energy expenditure [31]. Furthermore, energy losses in the course of lung disease exceed patient’s dietary intake. In turn, poor nutritional status may be a subsequent considerable factor in the CF adult lifespan. This is confirmed by previous studies [21–25]. Research by Yen et al. [7] showed that higher body weight in childhood is associated with fewer pulmonary exacerbations and better survival through the first 18 years of life. According to Courtney et al. [26], BMI is not a reliable predictor of mortality but has a significant impact on the course of pulmonary disease. However, studies conducted by Sharma et al. [32] showed that wasting has a significant impact on the probability of survival in CF adult patients, regardless of lung function.

In this paper, we have shown the relationships between selected factors influencing disease progression and survival in adult CF patients. It is important, however, that all these factors interact with each other, creating the so-called vicious circle, which has already been shown in studies on a similar group of patients [9]. Therefore, it is important to take into account all the analyzed factors when monitoring the course of the disease in adult patients with cystic fibrosis.

## Conclusions

In summary, we can assume that all factors included in the study were significantly related to the survival rate of patients with cystic fibrosis. The shortest average life expectancy was indicated in people exhibiting severe mutation type, FEV_1_% < 40%, BMI < 18.5, and XDR or PDR *P. aeruginosa* prevalence. In comparison, among patients with lung function within the norm and BMI above 25, no one has died. Adult CF patients over 40 years of age, remained in groups of an unknown or mild type of mutation, with normal or moderate lung function, with no *P. aeruginosa*, or with strains susceptible to antibiotic treatment. Our study allowed us to determine that the period between 30 and 40 years of age is the most critical in CF adults’ life span. Furthermore, most exacerbations occurred over the period between 20 and 35 years of age.

## Data Availability

The datasets used and/or analyzed during the current study are available from the corresponding author on reasonable request.

## Abbreviations

CF: Cystic fibrosis
CFTR: Cystic fibrosis transmembrane conductance regulator
FEV_1_%: Predicted forced expiratory volume in 1 s (percentage of predicted value)
non-MDR: Non-multidrug resistant
MDR: Multidrug-resistant
XDR: Extensively drug-resistant
PDR: Pandrug-resistant
BMI: Body mass index
